# Nanophotonics out of equilibrium

**DOI:** 10.1515/nanoph-2024-0215

**Published:** 2024-05-06

**Authors:** Alejandro Manjavacas, Matthew Pelton, Matthew Sheldon, Maxim Sukharev

**Affiliations:** 16379 Instituto de Óptica (IO-CSIC), Consejo Superior de Investigaciones Científicas, Madrid 28006, Spain; Department of Physics, 14701 UMBC (University of Maryland, Baltimore County), 1000 Hilltop Circle, Baltimore, MD 21250, USA; 8788 Department of Chemistry, University of California, Irvine, 2123 Natural Sciences II, Irvine, CA 92697, USA; Department of Physics, Arizona State University, Tempe, AZ 85287, USA; College of Integrative Sciences and Arts, Arizona State University, Mesa, AZ 85212, USA

Much of optics – refraction, reflection, diffraction – involves light interacting with materials at, or nearly at, equilibrium. Even most nonlinear optics involves weak, perturbative interactions far from material resonances. On the other hand, absorption of a photon by a material excites an electron to a non-equilibrium excited state, typically setting off a series of ultrafast dynamical processes. Similarly, emission of a photon requires that an electron first be placed in an unstable excited state. A long-standing goal in nanophotonics has been to control these non-equilibrium processes by controlling the nanometer-scale structure of materials. This control has generally been achieved in two ways: (1) confining carriers in semiconductor nanostructures quantizes and modifies the available energy states for the carriers, tuning absorption and emission energies and modifying transition rates [1]; and (2) patterning structures that confine light locally changes the local density of electromagnetic modes, modifying absorption and emission processes [2]. If the coupling between emitters and confined optical modes is strong enough, spontaneous emission is no longer irreversible, and instead energy is coherently exchanged between the emitters and confined photons, forming new hybrid states known as polaritons [3]. One path to achieving such strong light–matter interactions is to confine light below the conventional diffraction limit by coupling to plasmon resonances in metal nanostructures [4]. Interaction of light with the metal nanostructures themselves creates a highly non-equilibrium electron population [5], [6], resulting in new photophysical and photochemical processes [7].

Five years ago, this journal surveyed the state of the art in nanophotonic control over non-equilibrium processes in a special issue on plasmon–exciton coupling [8]. Since then, the field has developed in several directions. Nanophotonic structures have been used to modify a wide range of processes beyond optical absorption and emission, including scintillation, photoemission of electrons, interaction of high-energy electrons with materials, optically induced magnetism, and resonant optical nonlinearities. Collective and non-classical phenomena in nanophotonic emitters have come to the fore, including superfluorescence and superradiance. It has been proposed that strong light–matter coupling can modify photochemical processes and may even modify the equilibrium potential-energy landscape for chemical reactions. Even as these new phenomena and applications emerge, key questions remain about the underlying processes. This Special Issue focuses on recent experimental and theoretical advances towards the required fundamental understanding of nanophotonic processes out of equilibrium.

Two Perspective articles exemplify these new directions. Russ and Eisler [9] review superfluorescence from arrays of semiconductor nanocrystals and thin films. This is a striking example of how light emission can be modified not only by controlling the structure of the materials but also by bringing them together to enable the spontaneous formation of coherence across multiple emitters. This coherence is an inherently non-equilibrium effect that leads to accelerated, coherent emission from the collection of emitters and may enable the development of quantum light sources. Delgado et al. [10] review light emission in the different regime of scintillation, where the emitter is excited by high-energy particles or ionizing radiation. Although excitation mechanisms and applications are different from those of fluorescence, the same approaches of controlling nanomaterial structure and engineering nanophotonic environments (plasmonic resonators, photonic crystals, and metasurfaces) promise similar benefits of improved efficiency, speed, and directionality of emission.

Continuing the theme of modifying emission through nanophotonic structures, Navarro-Barón et al. [11] provide a detailed theoretical investigation of how two-dimensional photonic crystals can modify the dipole radiation pattern of an emitter, focusing in particular on the potential for highly directional emission due to photonic van Hove singularities. Rather than using nanophotonic structures to enhance light emission, Martinez-Calderon et al. [12] use them to enhance electron emission; specifically, they examine the use of plasmonic structures on patterned metal surfaces to produce non-equilibrium “hot” electrons and thus enhance the efficiency of photocathodes as electron sources. Crampton et al. [13] also examine plasmon-induced photoemission; in their case, photoelectrons are excited by ultrafast laser pulses and imaged in a photoemission-electron-microscope (PEEM) configuration, enabling the propagation of surface plasmon polaritons to be monitored on nanometer length scales and femtosecond time scales.

As well as enabling photoemission, non-equilibrium electrons in metals can produce more complex optical phenomena. For example, González-Alcalde et al. [14] examine the inverse Faraday effect, in which a non-equilibrium magnetization is induced by light due to the circular motion of electrons in the metal, and show that it is significantly enhanced in metal nanostructures as compared to an unpatterned metal film. Silvestri et al. [15] develop a theory of resonant third-harmonic generation in metal films with refractive index near zero, a process driven by non-equilibrium electron dynamics.

These phenomena involve electrons within nanomaterials; Abad-Arrendondo and Fernández-Domínguez [16] examine instead the use of free electrons to induce excitations within nanophotonic materials. In particular, they model the interaction between a high-energy electron beam and a strongly coupled photon-exciton system, proposing a new way to excite and probe polaritons.

Continuing the study of polaritons, two papers examine strong coupling of molecular vibrations to cavities. Vibrational strong coupling has attracted a great deal of attention for the potential to modify ground-state chemical reactivity [17], but considerable uncertainty remains around reported results, and better fundamental understanding is required to resolve this controversy. Hirschmann et al. [18] and Yim et al. [19] both contribute to this understanding by examining the role of modes that are not directly involved in polariton formation. Hirschmann et al. study the relaxation from a coupled vibrational mode to an infrared-inactive vibrational mode, revealing the role of these “dark” vibrations in the dynamics of strongly coupled systems. Yim et al. focus on the interaction between an IR-active vibrational mode and the quadrupolar mode of a plasmonic metasurface, showcasing a method to harness these “dark” plasmonic states – ones that do not usually couple to light – and to use their small mode volumes to achieve strong localized coupling effects.

Together, the papers in this Special Issue illustrate the breadth and complexity of non-equilibrium processes in nanophotonics, making it clear that there is a rich range of phenomena remaining to be explored, controlled, and optimized for applications.
